# Phytoplankton Productivity in an Arctic Fjord (West Greenland): Estimating Electron Requirements for Carbon Fixation and Oxygen Production

**DOI:** 10.1371/journal.pone.0133275

**Published:** 2015-07-28

**Authors:** Kasper Hancke, Tage Dalsgaard, Mikael Kristian Sejr, Stiig Markager, Ronnie Nøhr Glud

**Affiliations:** 1 Nordic Center for Earth Evolution (NordCEE), Department of Biology, University of Southern Denmark, Odense, Denmark; 2 Greenland Climate Research Centre, Greenland Institute of Natural Resources, Nuuk, Greenland; 3 Arctic Research Center, Aarhus University, Aarhus, Denmark; 4 Institute for Bioscience, Aarhus University, Aarhus, Denmark; 5 Scottish Association for Marine Science, Scottish Marine Institute, Oban, United Kingdom; Mount Allison University, CANADA

## Abstract

Accurate quantification of pelagic primary production is essential for quantifying the marine carbon turnover and the energy supply to the food web. Knowing the electron requirement (*Κ*) for carbon (C) fixation (*Κ*_C_) and oxygen (O_2_) production (*Κ*_O2_), variable fluorescence has the potential to quantify primary production in microalgae, and hereby increasing spatial and temporal resolution of measurements compared to traditional methods. Here we quantify *Κ*_C_ and *Κ*_O2_ through measures of Pulse Amplitude Modulated (PAM) fluorometry, C fixation and O_2_ production in an Arctic fjord (Godthåbsfjorden, W Greenland). Through short- (2h) and long-term (24h) experiments, rates of electron transfer (ETR_PSII_), C fixation and/or O_2_ production were quantified and compared. Absolute rates of ETR were derived by accounting for Photosystem II light absorption and spectral light composition. Two-hour incubations revealed a linear relationship between ETR_PSII_ and gross ^14^C fixation (R^2^ = 0.81) during light-limited photosynthesis, giving a *Κ*_C_ of 7.6 ± 0.6 (mean ± S.E.) mol é (mol C)^−1^. Diel net rates also demonstrated a linear relationship between ETR_PSII_ and C fixation giving a *Κ*_C_ of 11.2 ± 1.3 mol é (mol C)^−1^ (R^2^ = 0.86). For net O_2_ production the electron requirement was lower than for net C fixation giving 6.5 ± 0.9 mol é (mol O_2_)^−1^ (R^2^ = 0.94). This, however, still is an electron requirement 1.6 times higher than the theoretical minimum for O_2_ production [i.e. 4 mol é (mol O_2_)^−1^]. The discrepancy is explained by respiratory activity and non-photochemical electron requirements and the variability is discussed. In conclusion, the bio-optical method and derived electron requirement support conversion of ETR to units of C or O_2_, paving the road for improved spatial and temporal resolution of primary production estimates.

## Introduction

Photosynthesis is the fundamental biological process that converts inorganic carbon into living biomass from solar radiant energy. Through photosynthesis, microalgae primary production fuels the marine food web and its efficiency and dynamics influence the energy supply to higher-trophic levels [1]. Understanding the conversion of the photon flux energy from solar radiation towards fixation of inorganic carbon (CO_2_) and/or production of oxygen (O_2_), forms the basis for quantifying the primary production.

Conventionally, O_2_ production, ^14^C and ^13^C fixation, techniques have been used to quantify either gross (GPP) or net (NPP) primary production [2–4]. It is, however, still debated what the methods really measures and how to arrive at accurate gross or net primary production rates [5–8]. Traditionally, GPP refers to the fixation of inorganic ^14^CO_2_ without accounting for any carbon (C) losses to respiration, while NPP refers to the ^14^CO_2_ fixation after subtracting the respiratory CO_2_ ‘lost’ by phytoplankton over a diel cycle [2,9]. Conventionally, it is assumed that short time (1–2h) incubations yield estimates of GPP while NPP is obtained over 24h incubations [10]. However, Williams et al. [7] convincingly showed that 2h incubations can produce NPP estimates, a conclusion supported by Pei and Laws [5]. Productivity can also be measured from a net change in O_2_ concentration over a diel cycle (24h). This way the measure includes the respiratory O_2_ consumption of the heterotrophic community of the sample including phytoplankton itself and is defined as the Net Community Production (NCP) [10]. In many marine systems, including the Arctic, low phytoplankton biomass limits the application of ^14^C and ∆O_2_ techniques to longer incubation times, i.e. 24 hours. And as of today, marine primary production estimates are primarily based on discrete bottle measurements of GPP or NPP with a limited spatial and temporal resolution, with an unquantified degree of uncertainty and the risk of bottle effects [6,11].

Pulse Amplitude Modulated (PAM) fluorescence [12] or Fast Repetition Rate fluorometry (FRRf) [13,14] provide a non-invasive and fast assessment of the conversion of the photon flux to a rate of electron transfer (ETR) in Photosystem II (PSII). Such variable fluorometry methods can be applied *in situ* and represent an alternative measuring approach for photosynthetic activity in phytoplankton. Variable fluorescence can provide a high temporal (seconds) and spatial resolutions compared to traditional bottle incubations. Thus, if ETR can be converted to GPP or NPP based on an adequate understanding of the intermediate processes and on empirical evidence, variable fluorescence can be applied for primary production estimates in absolute terms [15]. Such knowledge enables the assessment of primary productivity with a high temporal resolution, and potentially enables the use of moorings and glider platforms for efficient and large-scale assessment of marine primary productivity.

Conversion of ETR to C fixation or O_2_ production is, however, still challenging [15–17]. The relationship between ETR and C fixation/O_2_ production has been compared in a range of studies on algal cultures and pelagic ecosystems and generally linear correlations are documented between ETR and gross C fixation and/or O_2_ production [18–21]. Deviations are reported under extreme conditions as for instance very high or low light conditions [22,23], extreme temperature [22,24], or nutrient stress [25,26]. Discrepancies have been proposed to be caused by changes in O_2_ consumption in the light, cyclic electron transport around PSII and I, Mehler-type reactions, and electron requirements for nutrient uptake and cellular maintenance. In some studies the interrelations between ETR and C fixation/O_2_ production have also been shown to be species-specific [20,24,26].

Lately, focus has been increasingly directed towards deriving the electron requirement for photosynthesis [15,17,20,21]. Lawrenz et al. [15] compiled a large amount of ETR data obtained using FRRf instruments and compared them to available ^14^C uptake rates across different regions. They arrived at a mean electron requirement for carbon fixation of 10.9 ± 6.9 mol é (mol C) ^−1^, overall ranging from 1.2 to 54.2 mol é (mol C)^−1^. The large variability partly originates from the multiple experimental approaches included in the study and the varying accuracy in the assessment of the light absorption by PSII. Still only few studies have focused on deriving the electron requirement for carbon fixation and oxygen production applying PAM fluorescence [21], none including both short and long term incubations. Comparisons of PAM versus FRRf measurements have shown a close relationship between the two, but with FRRf overestimating primary production relative to PAM measurements [21]. Essential for the conversion of ETR to absolute rates of primary production is an accurate assessment of the PSII-specific light absorption and of the available spectral irradiance. Only few studies have sufficiently included this when PAM derived quantum yields are converted to absolute units of ETR [17,21].

In the present study, we investigated the relationship between photosynthetic electron transport rate, ^14^C and ^13^C fixation, and O_2_ production of the natural phytoplankton community in the inner and outer part of an Arctic fjord. The aim was to quantify the electron requirement for gross and net carbon fixation and NCP in a natural low-biomass pelagic ecosystem. Through careful assessment of the PSII-specific light absorption and incubator spectral irradiance, absolute rates of ETR were derived and compared to measured rates of C fixation and O_2_ production. Variability of the electron requirement and photosynthetic efficiency is discussed along with the potential for applying PAM fluorescence for assessing *in situ* productivity in marine systems.

## Theory

In this section, we present how ETR and GPP can be calculated in absolute terms from PAM measurements when combined with knowledge of the absolute rate of photons absorbed by photosystem II.

Gross photosynthesis from variable fluorescence (P_PSII_) can be quantified in absolute units of C (P_PSII_C_, mmol C L^−1^ s^−1^) or O_2_ (P_PSII_O2_, mmol O_2_ L^−1^ s^−1^) from the knowledge of the quantum yield of charge separation in PSII (Φ_PSII_), the spectrally-weighted specific absorption of photons in PSII ($\bar{a}$_PSII,_ m^−1^), the integrated incident irradiance (E_PAR_), and the electron requirement for C or O_2_ (*Κ*_X_), respectively (Eq 1) [27,28].

$$P_{PSII\_X}=\Phi_{PSII}\times {\bar{a}}_{PSII}\times E_{PAR}\times \frac{1}{K_{X}}$$ (1)

**Fig 1:**
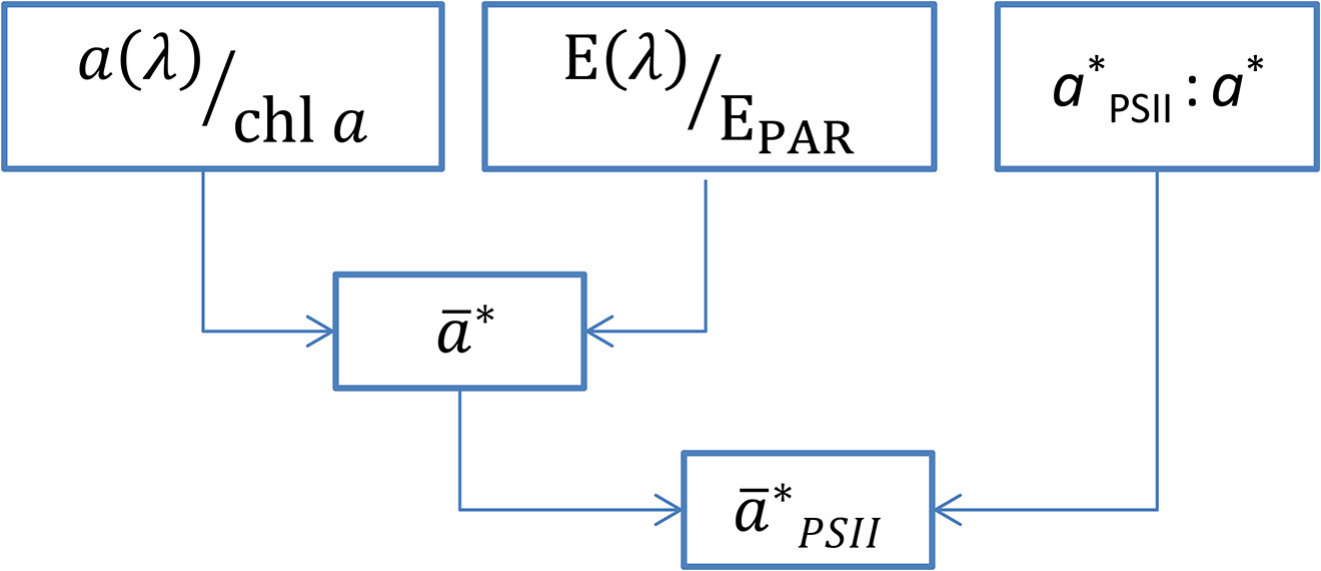
Schematics of the bio-optical approach used to quantify the rate of photons absorbed in photosystem II. First, the phytoplankton absorption spectrum (*a*(λ)) is normalized to the chl *a* concentration [a*(λ)]. Then, a*(λ) is weighted to the spectrum of the incubation light [E(λ)/E_PAR_] to give the spectrally-weighted chl *a*-specific light absorption ($\bar{a}*$). Finally $\bar{a}*$ is corrected for the ratio of absorbed quanta in PSII (a*_PSII_:a*) to yield the PSII-specific light absorption coefficient [$\bar{a}*$_PSII_, m^2^ (mg chl *a*)^−1^]. See text for details.

Where E_PAR_ is the integrated Photosynthetic Available Radiation between 400 and 700 nm, and *Κ*_X_ is the electron requirement for carbon fixation (*Κ*_C_) or oxygen production (*Κ*_O2_), respectively, in units mol electrons (mol C fixed or O_2_ produced) ^−1^. Note, that the spectral distribution of EPAR, i.e. E(λ), is here included in $\bar{a}$_PSII_ (see below, Fig 1). By normalizing $\bar{a}$_PSII_ to the Chlorophyll *a* (chl *a*) concentration [denoted $\bar{a}*$_PSII_] productivity is given in units per chl *a*, which is convenient for comparing rates across different environments and biomass abundances (Eq 2).

$${P^{*}}_{PSII\_X}=\Phi_{PSII}\times {\bar{a}}_{PSII}^{*}\times E_{PAR}\times \frac{1}{K_{X}}$$ (2)

Here we introduce the symbol *Κ* (Greek Capital letter Kappa) for the electron requirement for C fixation (*Κ*_C_) or O_2_ production (*K*_O2_), respectively. Previously in the literature, the symbol Φ_e,C_ has been used for the electron requirement for carbon fixation [15,21]. However, in photosynthetic research most often Φ refers to a quantum yield of a process [mol product (mol photons absorbed)^−1^], being the reciprocal of the quantum requirement [mol photons (mol product)^−1^]. Thus, we consider using Φ to be counterintuitive and instead prefer applying the symbol *Κ* to describe the electron requirement for photosynthetic carbon fixation or O_2_ production.

The quantum yield of charge separation in PSII (Φ_PSII_), also often named the quantum efficiency of photosynthesis, can be measured from variable fluorescence, e.g. using a Pulse-Amplitude-Modulated (PAM) fluorometer that measures the conversion efficiency of photons to electrons in PSII [12]. Measurements of the quantum efficiency rely on measuring the ratio between the minimum and maximum PSII fluorescence before and after a saturation pulse, which is why the quantum efficiency arrives on a biomass independent scale between zero and one; one being the theoretical maximum value given that all photons absorbed by PSII yields an electron transport. Typically the maximum quantum yield for marine algae is 0.5 to 0.8 and species dependent [24,29].

The absorption of photons in PSII [$\bar{a}$*_PSII_, m^2^ (mg chl *a*)^−1^] can be quantified using a spectrally-resolved bio-optical approach, combining information of the phytoplankton absorption spectrum [*a*(λ), m^−1^], the chl *a* concentration [chl *a*, mg m^−3^], the spectral light quality [E(λ)/E_PAR_, dimensionless], and the absorption ratio of PSII to the total absorption [a*_PSII_: a*, dimensionless] (Fig 1) [27].

First, *a*(λ) can be determined using standard spectrophotometric methods [30,31]. After normalization to chl *a*, a*(λ) is spectrally weighted to the spectrum of the light source [E(λ)/E_PAR_] according to Eq 3 [32,33].

$$\bar{a}*\;=\frac{\left[\sum_{400}^{700}a*\;\left(\lambda \right)\times E\left(\lambda \right)d\lambda \right]}{E_{PAR}}$$ (3)

Secondly, the ratio of absorbed quanta in PSII (a*_PSII_: a*) can be obtained from measuring the fluorescence excitation spectrum [34] and scale it to a*(λ), by applying the ‘non-overshoot’ procedure [27,35]. This procedure quantifies the spectral absorption in PSII, which includes the light absorption by light-harvesting pigments associated with PSII but excluding photo-protective carotenoids and light-harvesting pigments associate with PSI. Obtaining the phytoplankton fluorescence excitation spectrum is, however, cumbersome and requires a scanning spectro-fluorometer with is not available to most research laboratories. A simple alternative to determining the PSII-specific absorption is to multiply $\bar{a}*$ with the fraction of quanta absorbed in PSII to the total absorption (a*_PSII_: a*) (Fig 1). This procedure is applied here. It requires the knowledge of the ratio a*_PSII_: a*, which is specific to phytoplankton pigment-classes, but can be found in a comprehensive investigation of 33 species of phytoplankton representing 12 pigment classes [36].

Finally, the PSII-specific absorption coefficient (*a*_PSII_*) is calculated from Eq 4.

$${\bar{a}}_{PSII}^{*}\;=\bar{a}*\times (a_{PSII}^{*}:a*)$$ (4)

If excluding *Κ*_X_ from Eq 2 the equation calculates the electron transfer rate through PSII in absolute units (Eq 5, ETR_PSII_, mol é (mg chl *a*)^−1^ time^−1^).

$${{ETR}^{*}}_{PSII}\;=\Phi_{PSII}\times {\bar{a}}_{PSII}^{*}\times E_{PAR}$$ (5)

Here we aim to quantify *Κ*_C_ and *Κ*_O2_ from measures of P*_PSII_C_ and P*_PSII_O2_. Thus reorganizing Eq 2 and 5, the electron requirement can be calculated (Eq 6):

$$K_{X}=\frac{{ETR}_{PSII}^{*}}{{P^{*}}_{PSII\_X}}$$ (6)

In the following, we estimate ETR*_PSII_ in absolute units [mmol é (mg chl *a*)^−1^ h^−1^] and the rates of O_2_ production and C fixation to derive the electron requirement for O_2_ production and C fixation, respectively.

## Materials and Methods

### Study area and experimental setup

Water was sampled in the Arctic Godthåbsfjord in West Greenland, during August-September 2013. Samples were taken at two stations, GF3 and GF7 representing the outer and inner part of the fjord system, respectively. The two stations are part of the marine monitoring program MARINBASIS maintained by the Greenland Institute of Natural Resources and the University of Aarhus (www.nuuk-basic.dk). Water was sampled in the euphotic zone at 5 and 20m depth with a 5L Niskin water sampler, screened through a > 280 um mesh to remove large zooplankton and transported to the laboratory within 1 to 2 hours. In total ~120L of water was sampled at each station. *In situ* profiles of conductivity, temperature, depth (CTD), chl *a* fluorescence, optical turbidity and downwelling spectral irradiance were obtained at each station using a free-falling Optical Profiler II (Satlantic, Halifax, Canada). Geographical position, sampling date, *in situ* water temperature, salinity and light availability are given in Table 1.

**Table 1:** Geographical and water column data for sampled stations.

Stations	Location	Sampling	Depth	*In situ* temp	Salinity	E_PAR_/E_0_	NH_4_^+^	NO_3_^−^	PO_4_^3–^
#	Lat., Long.	date	m	°C	PSU	%	μM	μM	μM
GF3	N64°07′ W51°53′	29.08.13	5	4.0	31.4	40 (480)	0.35	4.50	0.45
			20	3.7	32.6	2.7 (32)	0.20	5.00	0.45
GF7	N64°26′ W51°31′	02.09.13	5	4.0	30.4	39 (468)	0.30	1.00	0.10
			20	3.8	31.8	2.2 (26.4)	0.40	2.00	0.25

Geographical position, sampling time, *in situ* condition and nutrient concentrations at the sampled stations. Light at depth is given as percent of bright surface irradiance (E_0_ = 1200 μmol photons m^−2^ s^−1^), and in absolute units in brackets (μmol photons m^−2^ s^−1^), calculated from the measured K_d_ (~0.18 m^−1^). The irradiance of the incubators was adjusted to correspond to the irradiance at 5 and 20 meters, respectively.

The concentration of NO_3_^−^+NO_2_^−^,collectively termed NO_3_^−^, was determined as NO on a NOx analyzer (Model 42C, Thermo Environmental Instruments Inc.) after reduction to NO in hot Vanadium Chloride [37]. PO_4_^3–^ and NH_4_^+^ were determined by standard colorimetric methods [38] on a Shimadzu UV-1800 spectrophotometer. Concentrations are given in Table 1.

In the laboratory water samples were incubated at light-limited (~40 μmol photons m^−2^ s^−1^) and light-saturated (~500 μmol photons m^−2^ s^−1^) conditions in two large water bath incubators (100 x 100 x 15cm) kept close to *in situ* temperature (6°C) by a thermostat controlled heater, both installed inside a cooling container (~2°C). The large surface area of the incubator ensured a homogeneous illumination by halogen light sources of incubated bottles for ^13^C and ^14^C fixation, O_2_ production and ETR_PSII_. The specific scalar irradiance (E_PAR_) of each incubated bottle was measured using a small 4π scalar irradiance sensor connected to a light meter (ULM-500, Walz). The spectral composition of the incubator light was measured using the surface unit from the Satlantic Optical Profiler II. The specific irradiance and the spectral composition of the incubator light were used for further calculations of the light absorption by phytoplankton, see below. Twenty-four hour incubations were performed under an 8:8:8 hour light:dark:light regime to mimic the natural light conditions with a day length of ~16 hours (and an ~8 hours night period), as incubations were started around noon. In addition, the approach avoided light exposure times of >8 hours (discussed below).

### Bio-optics, chl *a* and light microscopy

Optical densities of total particulate matter (OD_t_, 300–800 nm) was measured from 1 liter of sea water filtered onto GF/F glass fiber filters (Whatman Inc., Florham Park, NJ, USA) in a spectrophotometer (Shimadzu UV‐2401PC UV‐Vis) equipped with an integrating sphere (ISR‐240A) as described by Staehr and Markager [39]. Triplicate filters were used and each filter was measured three times in order to minimize noise. Values above 750 nm were subtracted. Total spectral absorption of suspended particles was obtained by the scattering correction method [40].

$$a_{t}\left(\lambda \right)=2.303\times S\times {OD}_{filt}\left(\lambda \right)\times \left[0.378+0.523\times {OD}_{filt}\left(\lambda \right)\right]/V$$ (7)

where *a*_t_ is the total absorption coefficient of particles, S is the clearance area of the GF/F filter (m^2^), V is the filtered volume (m^3^) and [0.378 + 0.523 × OD_filt_(λ)] is the beta-factor correcting for a longer path length in the filter compared to suspension. The in vivo phytoplankton absorption spectrum [*a*_ph_(λ), m^−1^] was then determined according to [30]:

$$a_{ph}\left(\lambda \right)=a_{t}\left(\lambda \right)-a_{NAP}\left(\lambda \right)$$ (8)

Where *a*_NAP_ is the absorption coefficient of non-algae particles, i.e. detritus and the non-pigmented parts of phytoplankton, after extraction in methanol. The chl *a*-specific absorption was then calculated from [31]:

$$a*\left(\lambda \right)=a_{ph}\left(\lambda \right)/\left[chl\;a\right]$$ (9)

The spectrally weighted chl *a*-specific absorption ($\bar{a}$*, m^2^ (mg chl *a*)^−1^), and the spectrally weighted PSII-specific absorption ($\bar{a}$*_PSII_) were calculated from Eqs 3 and 4, respectively. The Chl *a* concentration (mg m^−3^) was measured from 300 mL of sea water filtered onto GF/F glass fiber filters (Whatman) extracted in 10 mL 96% ethanol during 24h (dark, 4°C). The concentration was determined in triplets using a pre-calibrated fluorometer (Turner Designs TD-700).

Water samples were collected for phytoplankton cell count and species identification, and were fixed with LUGOL (neutral, 1% final solution). The samples were analyzed in a light microscope using a Palmer Maloney chamber (0.1 mL) and after filtering through a 0.45 um polycarbonate filter (50 mL).

### PAM fluorescence

The quantum yield of charge separation in PSII (Φ_PSII_) was measured using a PhytoPAM variable fluorometer (System I, Walz, Effeltrich, Germany, Schreiber et al. [12]), equipped with a sensitive Photomultiplier-Detector (PM-101P, Walz). Minimum (F_o_) and steady state (F_s_) fluorescence excitation was obtained using a weak and non-actinic modulated light supplied by a LED (light emitting diode, Array-Cone PHYTO-ML, Walz, Germany) during darkness or at the incubation irradiance, respectively. The maximum fluorescence (F_m_) was obtained during a red saturating light pulse (0.8s >1800 μmol m^−2^ s^−1^, Actinic LED-Array-Cone PHYTO-AL, Walz) ensuring that all PSII reaction centers were closed. The instrument excites fluorescence at four different wavelengths; however, in the present study we used data only from the red light excitation (665 nm), to exclude potential inter-sample differences in the light-harvesting pigments to chl *a* ratio. We use the nomenclature by van Kooten and Snel [41]. The maximum quantum yield of charge separation (Φ_PSII_max_) was calculated according to Eq 10 [42] after subtraction of the blank fluorescence, measured from a 0.2 μm filtered water sample:

$$\Phi_{PSII\_max}=\left(F_{m}-F_{0}\right)/F_{m}$$ (10)

Under actinic illumination, the operational quantum yield (Φ_PSII_) was calculated from the steady-state fluorescence (F_s_) and the maximum fluorescence after a saturation pulse (F_m_’), by replacing F_o_ and F_m_ with F_s_ and F_m_’, respectively in Eq 10. When Φ_PSII_ was quantified in samples incubated in the water bath a subsample was transferred to the PAM cuvette and measured within a few seconds. PAR inside the PAM cuvette was adjusted to match PAR of the incubator for each step of the light gradient after measuring the irradiance with a 4π scalar irradiance sensor and light meter (ULM-500, Walz). The spectral composition of the PAM cuvette incubation light was measured using the surface unit of the Satlantic Optical Profiler II.

ETR versus irradiance (P-E) curves were calculated from the instantaneous quantum yield in samples after 2 hours of incubation in 100 mL Winkler bottles, in a light gradient. The quantum yield was measured in the exact same bottles from which ^14^C fixation was measured (see below). The P-E curves were fitted from Eq 11 [43], as no photoinhibition was observed. The maximum photosynthetic rate (P_max_), the light utilization coefficient (α) and the light saturation index (E_k_) was calculated; E_k_ = P_max_/α. Curve fitting was carried out using ordinary least-squares criterion in Origin 8.5 (OriginLab).

$$P=P_{max}\times \left[1-e^{\left(-\alpha \times E_{PAR}/P_{max}\right)}\right]$$ (11)

### ^14^C fixation

Gross ^14^C fixation rates were measured in 100 mL Winkler bottles in a light gradient incubated for 2 hours (exact same as used for PAM P-E curves). We added 200 μl of Na_2_^14^CO_3_ with an activity of 20 μCi mL^−1^ [2]. The initial DIC concentration was assumed to be 2 mM according to the robust regional relationship between DIC and the salinity [44]. Nine bottles were incubated at different light intensities and 2 bottles in the dark. The content of all bottles was filtered onto GF/F filters that were transferred to glass scintillation vials, thereafter 100 μl 1 M HCl was added, and the filters were fumed for 8 hours. After addition of scintillation fluid (UltimaGold+) the samples were measured on a PerkinElmer scintillation counter. The dissolved fraction of fixed ^14^C was measured by collecting two duplicate 5 mL samples of filtrate from each Winkler bottle following the protocol by Moran et al. (2001). The dissolved ^14^C fixation was finally added to the particulate pool. The dark bottle activity was subtracted from the light bottles.

### ^13^C fixation

Diel rates of carbon fixation was measured from the ^13^C incorporation over a 24h time period according to Yun et al. [45], and normalized to the chl *a* concentration (P*_C_). Briefly, ^13^C bicarbonate was added to the sea water before incubation to a concentration of 200 μM in triplicate 500 mL square Nalgene polycarbonate bottles and incubated as described above. The initial DIC concentration was assumed to be 2 mM (see above). After the incubation the algae were filtered onto pre-combusted GF/F filters (Whatman). Filters were dried at 50°C for 48 hours before analysed for the content of ^13^C on a Thermo Elemental Analyser Flash EA 1112HT in line with a Thermo Delta Plus V isotope ratio mass spectrometer.

### O_2_ production

Diel rates of the net community production (P*_O2_, equal to NCP) were measured from the change of O_2_ concentration over a 24h time period under an 8:8:8 hour light:dark:light regime in the incubator. Incubations were done in five 100 mL glass Winkler bottles for each treatment (high and low light). For each incubation, five Winkler bottles were preserved with 1 mL 7 M ZnCl_2_ at the start and the incubated bottles were preserved after 24 hours incubation. The preserved bottles were stored in a water bath at ca. 5°C until analysis which was done within 24 hours of ZnCl_2_ addition.

Oxygen concentration was determined using a Clark type O_2_ microsensor [46] with a micro flow cell mounted on the tip. Water was drawn through the flow cell by gravity by keeping the surface of the source water 45 cm above the outlet. Once the flow path was filled with sample water the difference in pressure between inlet and outlet maintained a steady flow of 4 mL/min. The sample temperature was adjusted before entering the flow cell by passing through a 75cm coil of 1/16” steel tubing, that was positioned with the sensor and flow cell in a temperature controlled water bath (5.0°C ± 0.1°C). The O_2_ sensor was calibrated before and after the analysis of each sample. The calibration water was prepared as follows: Two liter of tap water was adjusted to within 50 μM of the expected sample O_2_ concentration and transferred to a gas-tight plastic bag [47], where after all bubbles were removed and 20 mL 7 M ZnCl_2_ was added to stop biological activity. The bag water was mixed and placed in the water bath to secure a stable temperature. The plastic bag was connected via a three way valve to the flow path upstream from the steel tubing coil. ‘Tygon’ tubing was used throughout this setup. During analysis the three way valve was turned every 120 seconds, alternating between drawing sample and calibration water though the flow cell on the O_2_ sensor, and the signal was read as the average of the last five seconds of each interval. The microsensor was connected to a Unisense PA2000 picoammeter and the signal was recorded using Unisense Sensortrace Basic and a Unisense ADC-216USB A/D converter.

## Results

### Algae composition and bio-optics

Light-microscopic analyses showed that the outer fjord station, GF3, was dominated by diatoms (*Chaetoceros sp*.) and smaller flagellates (*Cryptophyceae sp*. <15μm). The distributions between the two were 75:25% diatoms:flagellates in the surface (5m), and 15:85% in the lower euphotic zone (20m). The inner fjord station, GF7, was by far dominated by diatoms (>99%, *Chaetoceros sp*. and *Thalassiosira sp*.) with a minor presence of dinoflagellates (<1%, *Gymnodinium sp*. and *Scripsiella sp*.) at both 5 and 20 m. By classifying the phytoplankton in pigment groups based on their light-harvesting pigment signature, the fraction of absorbed quanta in PSII (a*_PSII_:a*) was derived, using the work by Johnsen and Sakshaug [36].

For diatoms, a*_PSII_:a* equals 0.76 and 0.74 for low and high light growth, respectively, whereas cryptophytes have a a*_PSII_:a* of 0.66 and 0.45 under low and high light, respectively [36]. Using these ratios we quantified a*_PSII_:a* for the sampled phytoplankton population (Table 2). The calculated a*_PSII_:a* ranged from 0.67 to 0.76 (dimensionless) and was used in the calculation of the PSII-specific light absorption (Eq 4).

**Table 2:** Bio-optical input parameters.

Station	Depth	Chl *a*	a*	a*_PSII_:a*	$\bar{a}*$	$\bar{a}*$	$\bar{a}*$	$\bar{a}*$_PSII_	$\bar{a}*$_PSII_	$\bar{a}*$_PSII_
					Water bath	PAM-HL^a^	PAM-LL^b^	Water bath	PAM-HL^a^	PAM-LL^b^
#	M	mg m^−3^	m^2^ (mg Chl *a*)^−1^	ratio	m^2^ (mg Chl *a*)^−1^	m^2^ (mg Chl *a*)^−1^	m^2^ (mg Chl *a*)^−1^	m^2^ (mg Chl *a*)^−1^	m^2^ (mg Chl *a*)^−1^	m^2^ (mg Chl *a*)^−1^
GF3	5	1.2 ± 0.4	0.0150	0.68	0.0108	0.0127	0.0158	0.0073	0.0086	0.0107
GF3	20	1.8 ± 0.1	0.0144	0.67	0.0099	0.0110	0.0149	0.0067	0.0074	0.0101
GF7	5	1.9 ± 0.1	0.0143	0.74	0.0097	0.0106	0.0146	0.0072	0.0079	0.0108
GF7	20	2.4 ± 0.1	0.0104	0.76	0.0068	0.0074	0.0106	0.0052	0.0057	0.0081

Chlorophyll *a* (chl *a*) concentrations (mean ± S.D.), chl *a*-specific absorption coefficient (a*), ratio of absorbed quanta in PSII (a*_PSII_:a*), spectrally weighted absorption coefficients ($\bar{a}*$), and the PSII-specific weighted absorption coefficient ($\bar{a}*$_PSII_) for the incubated samples and applied incubators (i.e. the water bath incubator and internal cuvette of the PAM instrument).

^a^ High Light

^b^ Low Light

**Fig 2:**
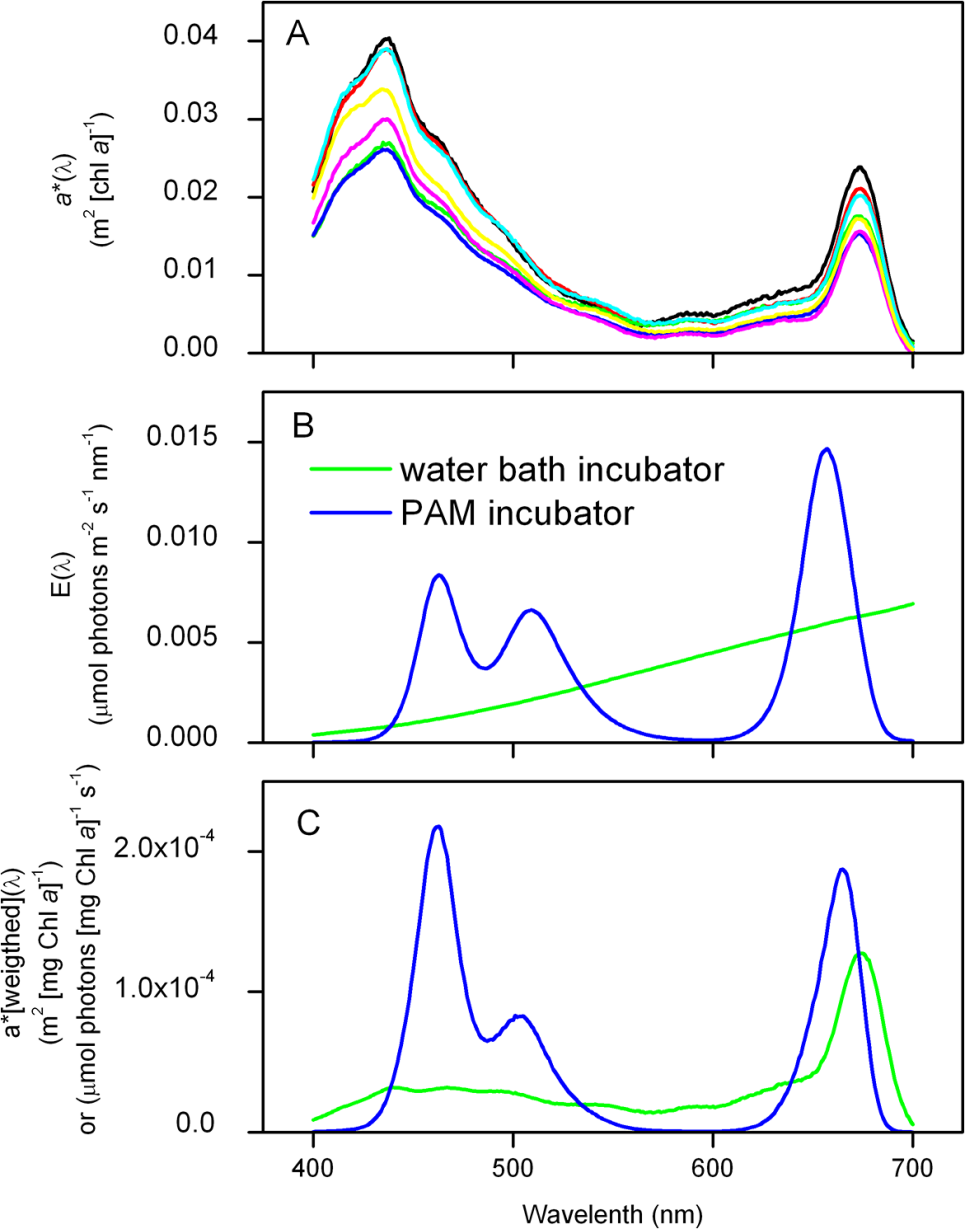
Variability in phytoplankton absorption spectra, incubator light quality and spectrally-weighted absorption. A) Chl *a*-specific in vivo absorption spectra [a*(λ)] at sampled stations and depths, B) spectral irradiance of the incubator light sources [E(λ)], and C) the spectrally-weighted chl *a*-specific absorption of phytoplankton at GF7 (5m), corrected for E(λ) in the water bath (green) and for the internal light source of the PhytoPAM (blue). Integrated values for a* and $\bar{a}*$ are given in Table 2.

Phytoplankton chl *a*-specific in vivo absorption coefficients (a*) ranged from 0.0104 to 0.0150 m^2^ (mg chl *a*)^−1^ demonstrating the natural variability of the light harvesting properties of the algae community, with chl *a* concentrations ranging from 1.2 to 2.4 mg m^−3^ (Table 2). The in vivo absorption spectra, along with the spectral scalar irradiance of the incubator light source (E(λ)/E_PAR_), and the derived spectrally weighted absorption spectra [$\bar{a}*$(λ)] are shown in Fig 2. Irradiance and weighted-absorption spectra are shown for both the incubator light source and the internal actinic light source of the PhytoPAM instrument (Fig 2B+2C). The absorption weighted to the internal PAM light source was used to calculate P_PSII_, while the weighted absorption from the incubator light was used to correct production rates comparing P*_PSII_ with P*_C_ and P*_O2_. The weighted PSII-specific absorption coefficients ($\bar{a}*$_PSII_) ranged from 0.0052 and 0.0108 m^2^ (mg chl *a*)^−1^ between samples and incubator light sources and are given in Table 2.

### Short-term incubations (2h)

**Fig 3:**
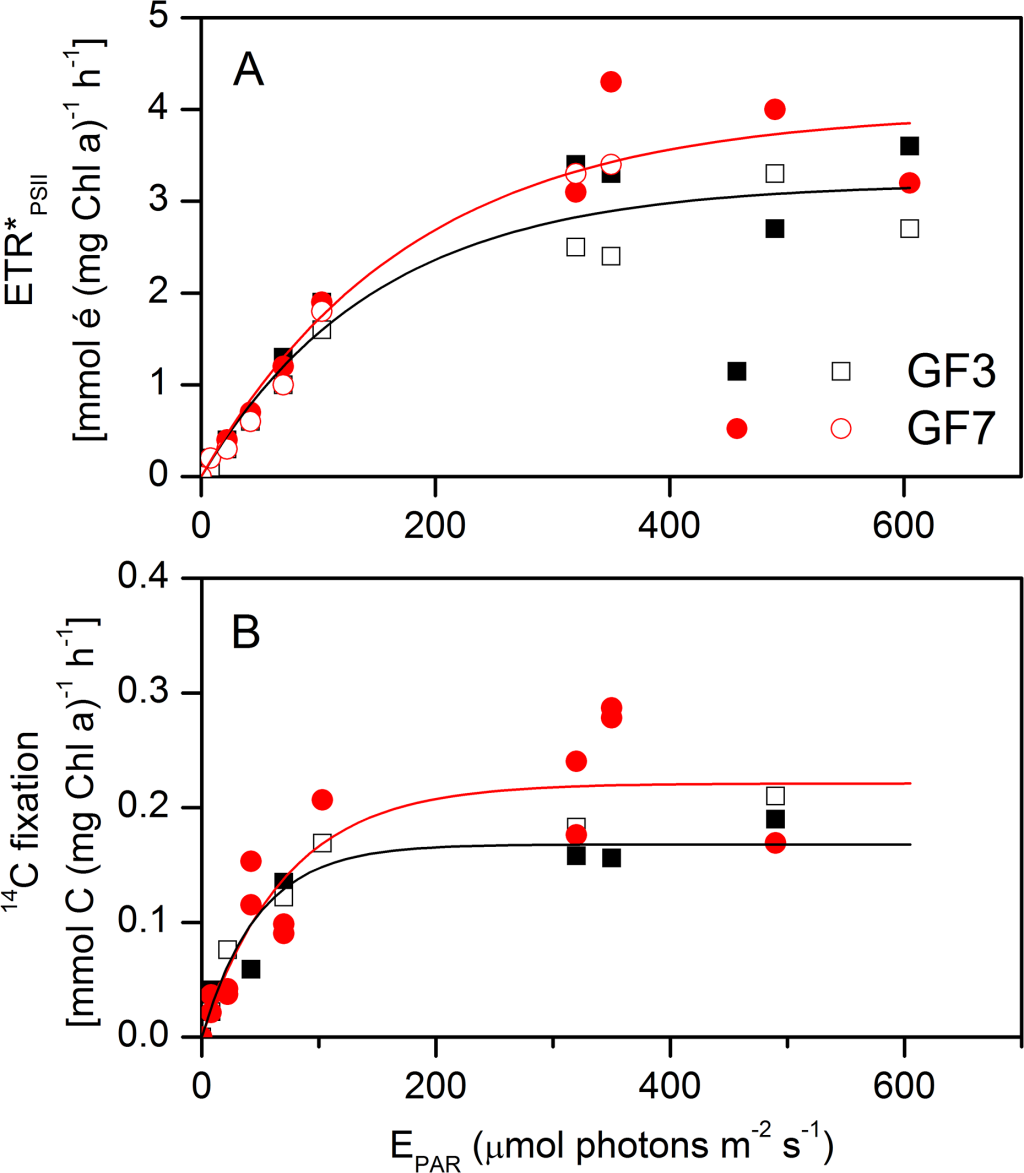
Photosynthesis versus irradiance (PE) curves of electron transfer and ^14^C fixation (2 hour incubations). A) Absolute electron transfer rates at PSII (ETR*_PSII_) derived from Eq 5, and B) measured ^14^C fixation (particular + dissolved fractions), as a function of E_PAR_ for GF3 and GF7, respectively. Lines are fitted with the Webb equation (Eq 11) for each station and photosynthetic parameters are given in Table 3. Measurements were duplicated for each station and the result is shown as open and closed symbol, respectively.

The electron requirement for gross ^14^C fixation was measured from simultaneous rates of gross electron transfer (ETR_PSII_, mmol é (mg chl *a*)^−1^ h^−1^) and gross ^14^C fixation (mmol C (mg chl *a*)^−1^ h^−1^) from the same bottles, incubated with surface water (5m) for 2 hours in an irradiance gradient (0 to 600 μmol photons m^−2^ s^−1^, Fig 3). The experiment was repeated two times for each of the two stations (no replicates). The P-E curves demonstrated minor differences between stations, but a somewhat different curvature of the relationship between methods, with a relatively steeper α for ^14^C fixation. The difference in curvature resulted in 2–3 times higher E_k_ for ETR_PSII_ than for ^14^C fixation. The photosynthetic parameters E_k_, P_max_ and α are given in Table 3.

**Table 3:** Fitting parameters for gross photosynthesis.

Station	Depth	Method	Pmax^a^	S.E.	alpha^b^	S.E.	R^2^	Ek^c^
#	m	#						
GF3	5	ETR	3.20	0.17	0.0215	0.003	0.95	149.2
GF7	5	ETR	3.98	0.26	0.0224	0.003	0.96	177.9
GF3	5	14C	0.17	0.02	0.0035	0.001	0.83	47.8
GF7	5	14C	0.23	0.02	0.0038	0.001	0.76	59.9

Photosynthesis versus irradiance (PE) parameters for 2h simultanous measurements of ETR_PSII_ and ^14^C fixation, derived from least square regression of the Webb equation (data in Fig 3, Eq 11).

^a^ Units of ETR*_PSII_ in mmol é (mg chl *a*)^−1^ h^−1^ and of ^14^C fixation in mmol C (mg chl *a*)^−1^ h^−1^

^b^ Units of ETR*_PSII_ in mmol é (mg chl *a*)^−1^ h^−1^ (μmol photon m^−2^ s^−1^) ^−1^ and of ^14^C in mmol C (mg chl *a*) ^−1^ h^−1^ (μmol photon m^−2^ s^−1^) ^−1^

^c^ Units in μmol photon m^−2^ s^−1^.

**Fig 4:**
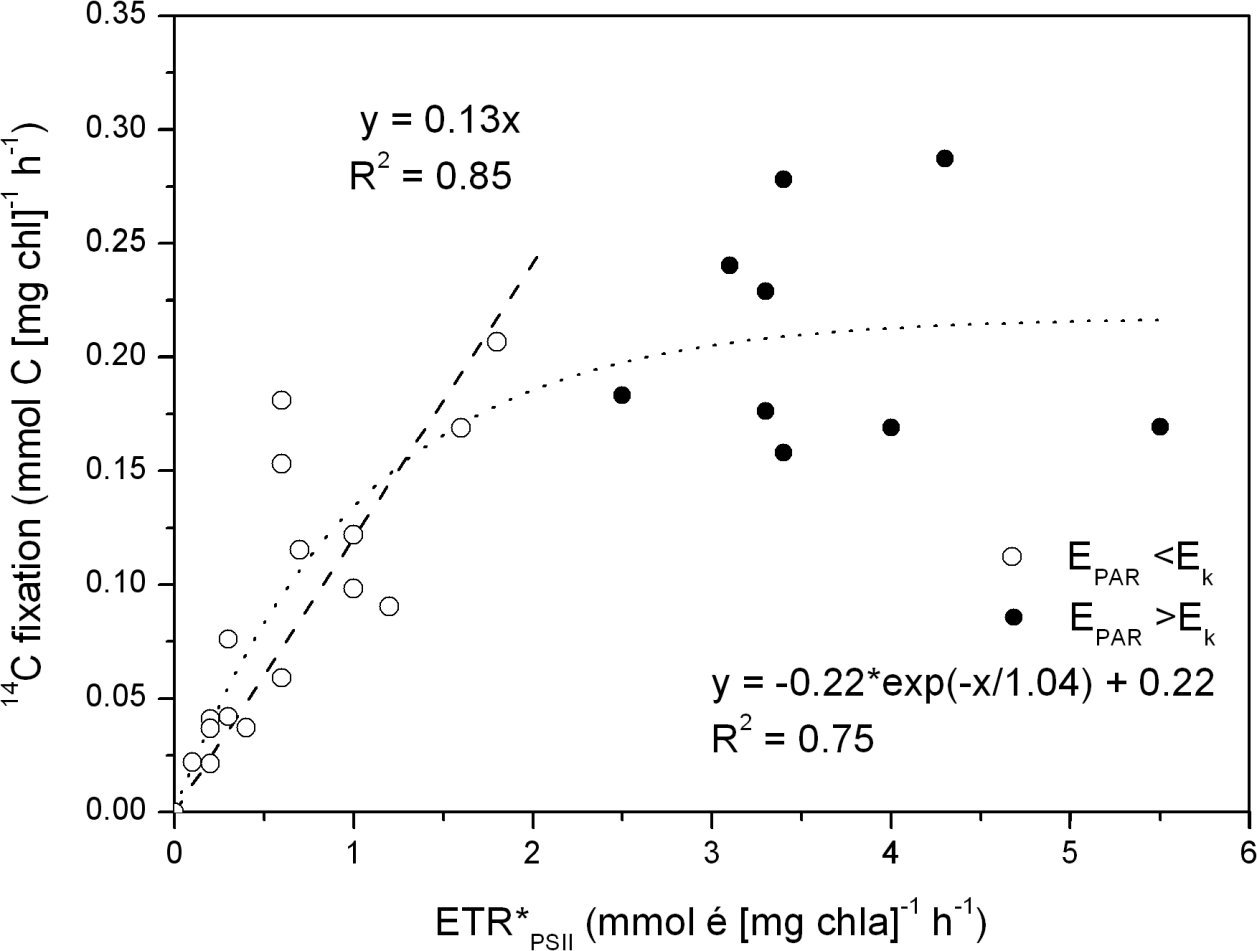
^14^C fixation versus electron transfer rate (ETR*PSII). ^14^C fixation rates versus absolute rates of ETR*_PSII_ based on four short-term (2h) incubation experiments in a light gradient (0–600 μmol photons m^−2^ s^−1^). The dashed line is a linear regression of data for E_PAR_ <E_k_ (open circles), and the slope coefficient represents the fixation of carbon to electron transfer in mol C (mol é)^−1^, corresponding to an electron requirement of 7.6 ± 0.6 mol é (mol C)^−1^. The complete data set expressed a non-linear relationship and is fitted with a simple inverted exponential decay function (dotted line, R^2^ = 0.75).

The short-term (2h) incubations revealed a significant linear relationship between C fixation and ETR_PSII_ during light-limited condition (E_PAR_<E_k_, corresponding to an ETR_PSII_ <2 mmol é (mg chl *a*)^−1^ h^−1^), giving a slope coefficient of 0.13 ± 0.014 mol C (mol é) ^−1^. This corresponded to an electron requirement for gross carbon fixation of 7.6 ± 0.6 mol é (mol C)^−1^ (R^2^ = 0.85, P <0.001, Fig 4). Inclusion of light-saturated samples lead to a non-linear relationship with ETR_PSII_ exceeding the ^14^C fixation under high light conditions (E>E_k_), that could be described applying a simple inverted exponential decay function (Fig 4). The equation parameters are given in the figure. The dissolved fraction of fixed ^14^C amounted to 18 ± 26% (data not shown) of the total and is accounted for in the assessments of the gross ^14^C fixation. In the following section we apply the electron requirement for the light-limited carbon fixation (i.e. 7.6 mol é (mol C)^−1^) to investigate the relationship between P*_PSII_, P*_C_ and P*_O2_ during 24h incubations.

### Variability of Φ_PSII_ and diel primary production (24h)

Electron requirement for net C fixation and NCP was investigated by applying long-term (24h) incubation experiments with a natural light-dark cycle. Triplicate samples from surface water (5m) and from the lower euphotic zone (20m) were incubated simultaneously, the former under light-saturated (E_PAR_ ~500 μmol m^−2^ s^−1^) conditions, and the latter under light-saturated and light-limited (E_PAR_ = 40 μmol m^−2^ s^−1^) conditions. These incubator irradiances correspond to the natural light intensities of a clear-sky day at the sampled depths (Table 1).

**Fig 5:**
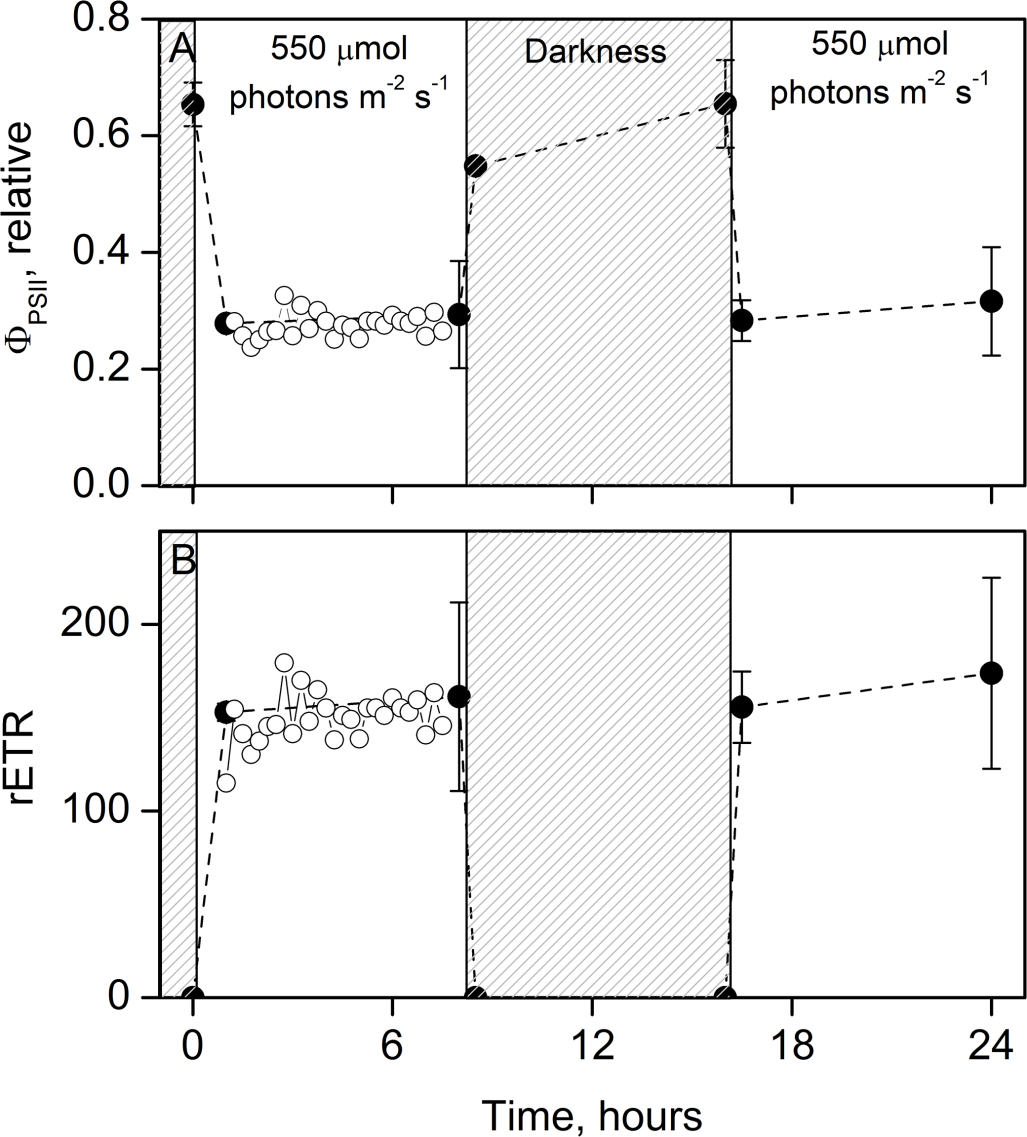
Variability of the photosynthetic efficiency during the 24h laboratory incubations. An example of (A) Φ_PSII_ as a function of incubation time during the 8:8:8 hour light:dark:light regime, and the (B) corresponding relative electron transfer rate (rETR = Φ_PSII_ × E_PAR_). Data are from GF7 5m incubated in the bath water (closed symbols, mean ± standard deviation, n = 3). Open symbols represent continuous measurements (every 15 min) of a subsample incubated inside the PAM fluorometer and shows the variability of Φ_PSII_ under stable conditions. The latter was stable over time and the slope coefficient was not statistically different from zero, ANOVA P >>0.05.

First, the temporal variability of Φ_PSII_ with incubation time was investigated over the light-dark cycle. The dark acclimated maximum Φ_PSII_ ranged from 0.55 to 0.65 while Φ_PSII_ was ~0.3 under high-light conditions (Fig 5A). Under low-light conditions (40 μmol photons m^−2^ s^−1^) Φ_PSII_ was ~0.6 and the maximum Φ_PSII_ was similar to the values at high-light (data not shown). Thus, Φ_PSII_ show the same trend and temporal variability during high and low light conditions. The response of Φ_PSII_ to a change from darkness to light, and verse versa, showed a fast acclimation response (<0.5h) and little variability during light hours (8h). The corresponding relative ETR (rETR) showed a steady electron generation in the light and obviously none during darkness (Fig 5B). The result demonstrated a stable ETR over time within the incubation period. As bottles for ETR, C fixation and O_2_ production were incubated simultaneously under the same conditions, it is reasonable to assume a linear relationship also for C fixation and O_2_ production rates during incubations [6,48].

**Fig 6:**
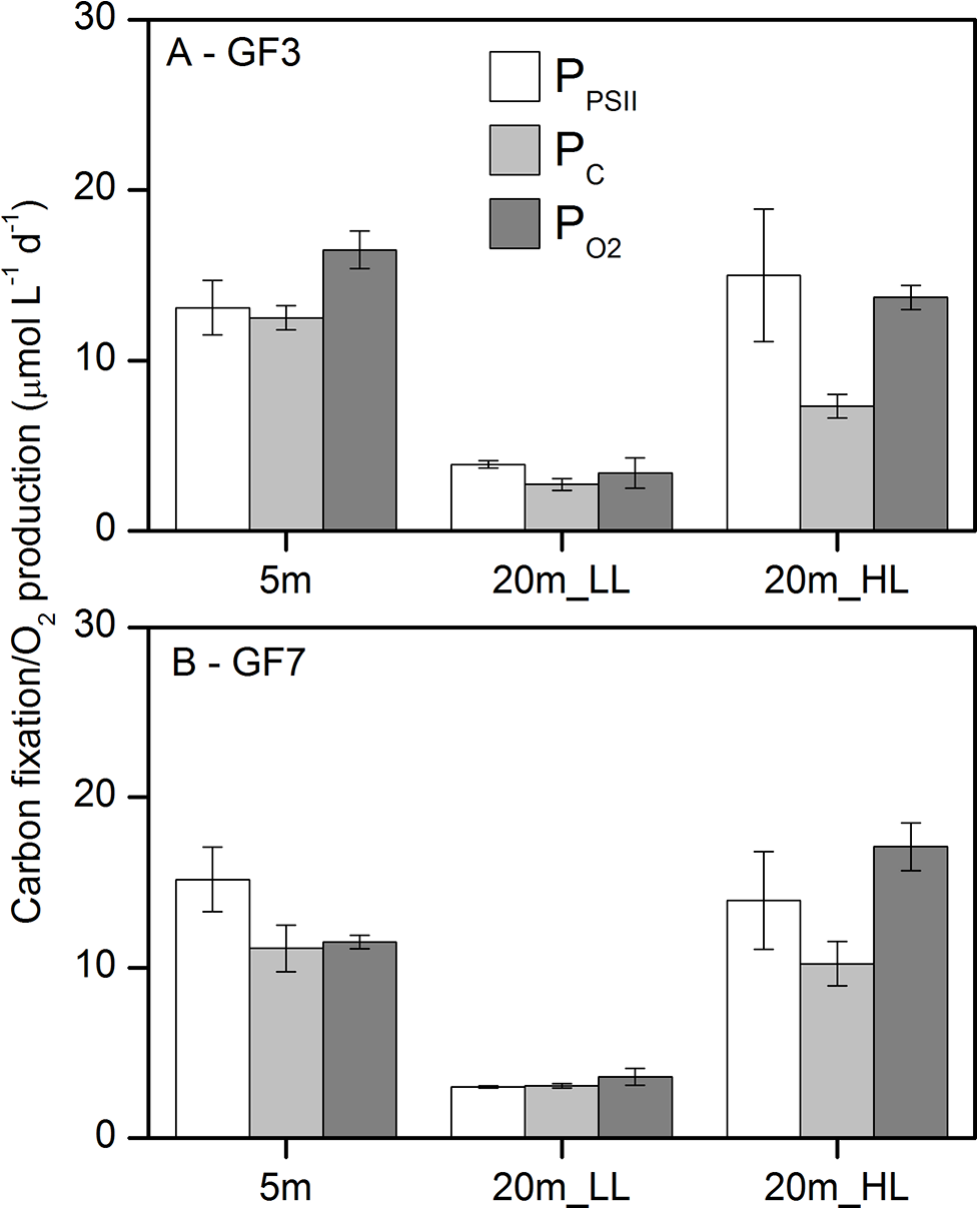
Comparison of diel rates of primary production derived from Φ_PSII_, ^13^C fixation and O_2_ production. Calculated rates of gross carbon fixation from Φ_PSII_ (P_PSII_), and rates of measured net ^13^C fixation (P_C_) and net O_2_ production (P_O2_) during 24h incubations with 16 hours of light, at (A) station GF3 and (B) GF7. P_PSII_ rates were calculated using the electron requirement for gross ^14^C fixation of 7.6 mol é (mol C) ^−1^ (Fig 4). Error bars for P_PSII_ are triplicate samples times 4 measurements across 24h (as shown in Fig 5), for P_C_ triplicate bottles, and for P_O2_ 5 replicate bottle incubations.

**Fig 7:**
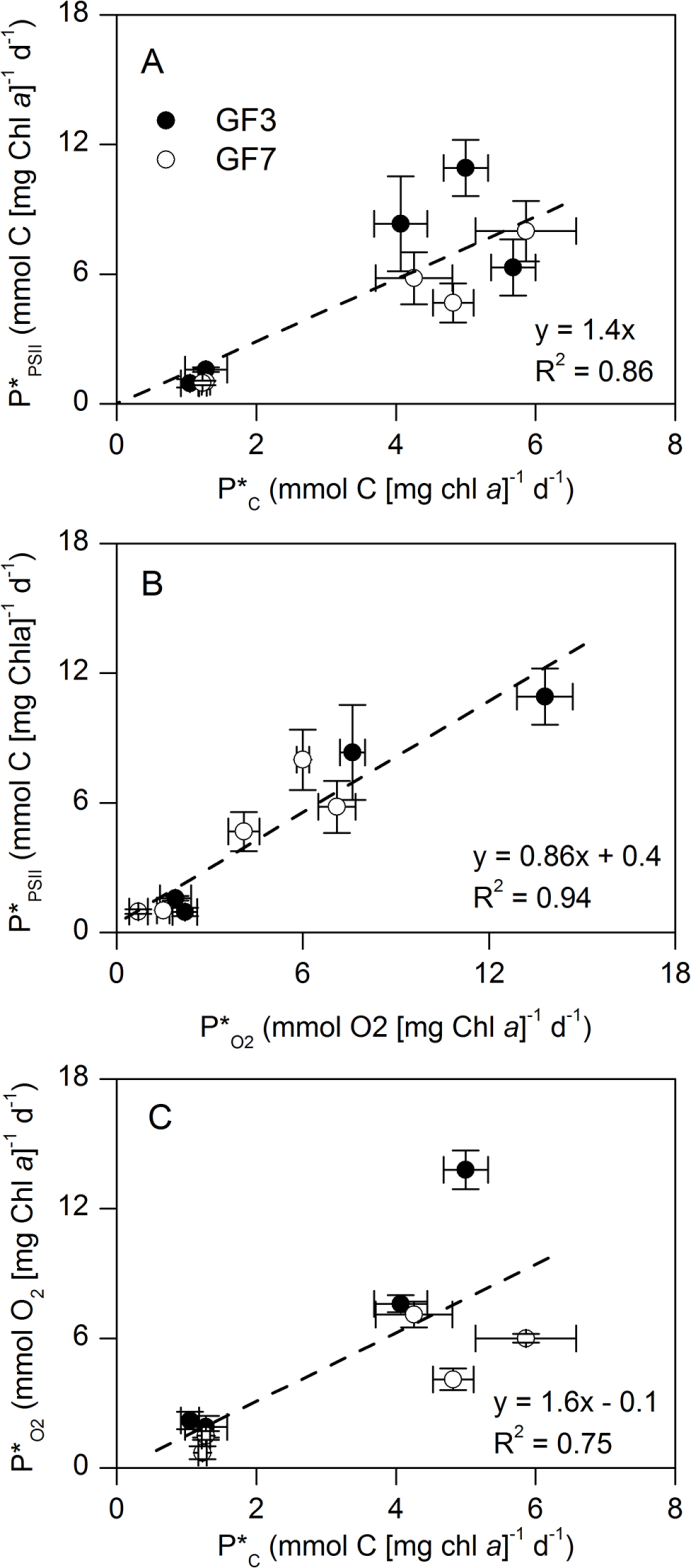
Relationships between diel rates of primary production derived from Φ_PSII_, ^13^C fixation, and O_2_ production. A) Gross carbon fixation from Φ_PSII_ (P*_PSII_) versus ^13^C fixation (P*_C_), B) P*_PSII_ versus net O_2_ production (P*_O2_), and C) P*_O2_ versus P*_C_ derived from 24h incubations with 16 hours of light (Fig 5). Data are pooled across depth, light intensity and stations (Fig 6). Rates are normalized to chl *a* and dashed lines are linear regressions, with A) forced through origo.

Diel primary production rates (μmol L^−1^ d^−1^) derived using the three methods are shown in Fig 6. The methods agreed well with one another at both 5 and 20m depths under both light-saturated and light-limited conditions, and between stations, with a minor suppression of P_C_ relative to P_PSII_ and P_O2_ at the 20m_HL treatment. The difference between methods was tested using two-sided paired t-tests between each method, and showed no significant difference between P_PSII_, P^C^ nor P_O2_ (P >0.05, performed using the build-in statistical routines in Origin 8.5, OriginLab). In detail, the difference between P_PSII_ and P_C_ were not significantly related to neither light intensity (P = 0.42, two-side t-test), water depth (P = 0.08) or station (P = 0.20). Neither was the difference between P_PSII_ and P_O2_ significantly related to light intensity (P = 0.21, two-side t-test), water depth (P = 0.45) or station (P = 0.20). Consequently, data were pooled across light intensity, depth and stations in order to quantify the relationship between P*_PSII_, P*_C_ and P*_O2_ (Fig 7). For this application the productivity was normalized to chl *a* to correct for the difference in biomass between depths and stations.

Fig 7A shows a linear regression between P*_PSII_ and P*_C_ with a slope coefficient of 1.4 ± 0.15 (mean ± SE, R^2^ = 0.86, P<0.001), which demonstrated a 1.4 times higher electron requirement for net carbon fixation than for gross carbon fixation. This implies a mean electron requirement for net C fixation of 10.9 ± 1.1 mol é (mol C)^−1^. Comparing P*_PSII_ and P*_O2_ demonstrated a slope coefficient of 0.86 ± 0.12 (mean ± SE, R^2^ = 0.94, P<0.001) corresponding to an electron requirement for net O_2_ production of 6.5 ± 0.9 mol é (mol O_2_) ^−1^ (Fig 7B). This is an electron requirement ~14% lower than for the gross C fixation

Plotting P*_O2_ versus P*_C_ yielded a slope coefficient of 1.6 ± 0.53 (mean ± SE, R^2^ = 0.75, P = 0.02) that demonstrated higher net O_2_ production rates than net carbon fixation rates across all samples (Fig 7C), i.e. the Photosynthetic Quotient (PQ).

## Discussion

In this paper, we estimate the electron requirement for C fixation and O_2_ production in phytoplankton in an Arctic fjord under post bloom conditions. Crucial to the calculation of ETR_PSII_ in absolute units is to quantify the amount of photons absorbed in PSII accurately. Hancke et al. [27] demonstrated a bio-optical approach to correct standard phytoplankton absorption measurements for the fraction of absorbed quanta in PSII [a*_PSII_:a*]. By weighting the absorption spectrum to the spectral quality of the incubator light source [35] they furthermore calculated and accounted for the PSII-specific absorption. Here we apply a simplified approach without using a sophisticated spectrofluorometer, but by applying published values for the fraction of PSII absorption determined for representative taxonomic groups [36]. By accounting for the phytoplankton light absorption and spectral irradiance of the incubator light (both of the waterbath incubator and inside the PAM cuvette), we calculate the PSII-specific absorption coefficient for the investigated communities at the applied conditions (Figs 1 and 2). This enables to correctly express ETR_PSII_ in absolute units and compare these to measured rates of C fixation and O_2_ production.

### Electron requirement for gross carbon fixation

Empirical evidence from a range of aquatic systems demonstrates a linear relation relationship between ETR and rates of C fixation and/or O_2_ production [20,21,49], however, deviation from linearity have also been reported [17].The derived electron requirement for primary production seem to express considerable variability and there still is considerable uncertainty about what causes this variability and how it relates to ‘true’ rates of primary production.

We found a consistent relationship between ETR_PSII_ and gross C fixation (Fig 4) yielding an electron requirement for gross C fixation of 7.6 ± 0.6 (mean ± SE, mol é (mol C) ^−1^) under light-limited conditions (E_PAR_<E_k_). There exists no exact theoretically defined requirement of electrons for C fixation. However, absolute ETR_PSII_ is considered a proxy for the gross photosynthetic rate, and the electrons generated at PSII are closely coupled to the gross O_2_ evolution rate from the water splitting complex in PSII [28]. Thus, it is theoretically given that the minimum electron requirement for gross O_2_ production, according to the standard Z-scheme of photosynthesis, is 4 electrons per O_2_ produced [28,49]. By multiplying this with a PQ of 1.4 produced O_2_ per fixed C [50], the minimum electron requirement for gross C fixation accounts to 5.6 mol é (mol C)^−1^. Here we found an electron requirement 1.4 times higher than this, which indicate an offset between ETR and C fixation. We speculate that this discrepancy is caused by alternative electron ‘requirements’ along the pathway from PSII to the fixation of carbon in the Calvin Cycle. Likely, these electrons are utilized to cover the energy requirement for nutrient uptake and cellular maintenance, and in Mehler-type reactions, especially at high light conditions [22,49].

Further, it is still debated whether short-term ^14^C fixation measurements represent NPP or GPP. Current consensus is that short term incubations (1 to 3h) quantify something intermediary to the strictly defined GPP and NPP. The uncertainties are mainly related to respiration of photosynthetically fixed ^14^C, recycling of respired ^14^CO_2_ and its preferential use over external CO_2_, but also growth rates have shown to impact the balance of NPP versus GPP [5–8]. Longer incubation periods and lower growth rates seem to bias short-term ^14^C fixation measurements towards representing NPP rather than GPP. For instance, rates measured during 2 hour incubations have shown to underestimate rates obtained during 60 min, and even more during 30 min [51,52]. In the current study we included the dissolved fraction of ^14^C labeled carbon to the gross C fixation measure, thus it is included in *Κ*_C_ for the gross C fixation. The fraction of dissolved to particular ^14^C uptake we here report are typical for pelagic C fixation [53,54].

For comparison to studies excluding a spectral correction for the incubator light spectrum, we recalculated the data of the present study excluding the spectral correction, which gave a mean *Κ*_C_ for gross C fixation of 11.2 mol é (mol C)^−1^, relative to 7.6 mol é (mol C) ^−1^ when spectrally corrected (Table 4). This gave an overestimation of *Κ*_C_ by 1.5 times and a 2-fold increase in the standard error, when excluding the spectral correction. The determined error imposed on *Κ*_C_ depends obviously on the spectral quality of the incubator light source and thus varies between studies. The closer the incubator light source is to a ‘perfect white light’ spectrum (also named a ‘flat’ spectrum) the lower is the error introduced in *Κ*_C._ In a field study, Kromkamp et al. [21] derived an electron requirement of 16.8 mol é (mol C)^−1^ across seasons from fresh water lakes (Table 4). In this estimate, they included the light absorption jointly for both PSII and PSI but did not correct for the spectral quality of the incubation irradiance. Assuming an equal distribution of the light absorption between PSII and PSI the comparable number to this study would be (16.8 × 0.5) 8.4 mol é (mol C)^−1^. This number is in close agreement with what found here, however theoretically it is overestimating *Κ*_C_ by the ratio of [a*_PSII_:a*] to 0.5. Kromkamp et al. estimated that correcting for the spectra difference between the red LEDs of the waterPAM actinic light and the natural underwater light field would reduce the estimated C fixation rate by 35%. Thus, the non-spectral corrected electron requirement [8.4 mol é (mol C)^−1^] would be overestimated by 35%.

**Table 4:** Mean values of the minimum electron requirement for primary production.

Method	Region/culture	Species	*Κ*_O2_	*Κ*_C_	Reference
PAM vs. O_2_/C			mol é (mol O_2_)^−1^	mol é (mol C)^−1^	
Same bottle, light gradient, PAM vs ^14^C (2h)	Arctic fjord	Natural phytoplankton community (diatom dominated, flagellates)	-	7.6+/-0.6 (3.5–11.7)	present study
			-	11.2+/1.3 (3.8–24.3)	present study, no-spectral correction
Separate bottles, low and high light, PAM vs O_2_ and ^13^C (24h)	Arctic fjord	Natural phytoplankton community (diatom dominated, flagellates)	6.5+/-0.9 (4.4–10.5)	10.9+/-1.1 (7.6–15.2)	present study
			9.2+/-1.4 (8.4–15.4)	15.9+/-1.6 (10.6–22.9)	present study, no-spectral correction
Separate bottles, PAM RLC (55s steps) vs. ^13^C in light gradient (4h)	Pure culture	*Pseudo-nitzschia pungens*	-	15.5 ^a^ ^,^ ^b^ (11.3–20)	Napoleon et al. (2013)[26]
		*Asterionellopsis glacialis*	-	19.2 (16.7–40.0)	
		*Heterocapsa sp*	-	9.2 (6.2–18.2)	
		*Karenia mikimotoı*	-	32.3 (25.0–46.2)	
Separate bottles, PAM RLC (55s steps) vs. ^13^C in light gradient (3h)	English Channel	Natural phytoplankton community (dominated by Diatoms and Dinophytes)	-	7.2^a^ (0.4–48.8)	Napoléon and Claquin (2012)[17]
Separate bottle, PAM PE (5min steps) vs. O_2_+^14^C PE light gradient (1h)	Pure culture	*Prorocentrum minimum*	4.5 (3.3–6.4)	6.2^c^ (3.8–7.6)	Hancke et al. (2008)[24]
		*Prymnesium parvum*	8.8 (5.5–11.8)	6.1 (4.8–8.2)	
		*Phaeodactylum tricornutum*	4.9 (3.6–6.3)	3.9 (3.3–4.8)	
Same bottle, PE curve (10min steps)	Pure culture	*Prorocentrum minimum*	3.3 (1.7–4.2)	-	Hancke et al. (2008)[27]
		*Prymnesium parvum*	7.3 (3.2–11.0)	-	
		*Phaeodactylum tricornutum*	3.8 (1.8–5.3)	-	
Separate bottles, PAM RLC (60s steps) vs. ^14^C light gradient (2h)	Freshwater lake	Cyanobacteria, chlorophytes	-	8.4^d^ (4.1–13.2)	Kromkamp et al. (2008)[21]
Same bottle, light gradient (4min steps)	Pure culture	*Chlorella vulgaris*	8.3 (8.1–8.4)	-	Wagner et al. (2006)[64]
		*Phaeodactylum tricornutum*	5.4 (4.5–6.3)	-	
Same bottle, PE curve (3min steps)	Pure culture	*Cylindrotheca closterium* (benthic diatom)	4.2^e^ (0.7–32)	-	Morris and Kromkamp (2003)[65]
Separate bottles, PAM RLC (90s steps) vs. ^14^C *in situ* (3h)	Freshwater reservoir	Diatoms, Chlorophytes, Cryptophytes	-	11.7 (5.1–19.8)	Gilbert et al. (2000)[66]
Same bottle, PE curve (10min steps)	Pure culture	*Chlorella vulgaris*	4.7^e^ (4.0–5.1)	-	Gilbert et al. (2000)[49]
		*Cryptomonas ovata*	3.7^e^ (3.5–4.3)	-	
		*Cyclotella meneghiniana*	3.8^e^ (3.5–4.0)	-	
		*Synechococcus leopoliensis*	4.4^e^ (3.0–5.3)	-	

Values (including the range) are derived for C fixation (*Κ*_C_) and O_2_ production (*K*_O2_) are from the present study and current literature by comparing corresponding rates of ETR, and C fixation or O_2_ production, respectively. The table includes only studies that estimate ETR_PSII_ from PAM measurements in absolute units thus considering the PSII specific absorption.

^a^ at E<Ek

^b^ only nutrient repleted cultures

^c^ across a temperature gradient (0–30°C). No trend with temperature

^d^ derived from 16.8 (8.2–26.4) times 0.5, assuming an equal distribution of the absorbed quanta between PSII and PSI

^e^ data from Suggest et al 2011[16]

Comparing *Κ*_C_ to previous studies is hampered by difference in measuring procedures and protocols that are used to quantify the absorption of photons in PSII (Eq 1). Table 4 provides values of *Κ*_C_ for the minimum electron requirement for C fixation and O_2_ production derived from the current literature. The table includes only studies that account for the PSII specific absorption, and attempt to express ETR in absolute units. Hancke et al. [24] found a mean *Κ*_C_ ranging from 3.9 to 6.2 mol é (mol C)^−1^ in mono cultures of different phytoplankton species, while accounting for the applied spectral irradiance and [a*_PSII_:a*] (Table 4). Somewhat higher values of *Κ*_C,_ ranging from 9.2 to 32.3 mol é (mol C)^−1^are derived from a similar study of Napoleon et al. [26], that accounted for [a*_PSII_:a*] but ignored the spectral quality of the incubator light.

Under high light intensities (E>>E_k_), we observed a non-linear relationship between ETR_PSII_ and the C fixation, (Fig 4). Even though many studies demonstrate a linear relationship, Napoleon and Claquin [17] report of a similar non-linear relationship between C and ETR at E_PAR_>E_k_, in a field study from the English Channel. They ascribed the non-linear relationship to alternative electron sinks caused by high irradiance, photo inhibition, Mehler-type reactions and nutrient uptake. In fact, Napoleon and Claquin [17] proposed an empirical algorithm to compensate for this. While the derived parameters apply well (R^2^ = 0.77) and the study appear thoroughly performed the derived parameterization has little theoretical foundation and cannot *a priory* be assumed to apply across seasons or regions.

### Electron requirement for net carbon fixation and O_2_ production

A challenge when comparing rates of ETR_PSII_ and NPP/NCP is to constrain the temporal variations in ETR during the incubation, as ETR is sampled instantaneously and C fixation or O_2_ production are integrated over the entire incubation. From monitoring ETR (or really Φ_PSII_) during a diel cycle with an 8:8:8 hour light:dark:light period we documented that ETR was stable under constant light, both at high and low light (Fig 5). This suggests that a single measurement after 1 to 2 hours under the desired light regime is sufficient to calculate the productivity for a 24h time period, under laboratory steady state incubations. Consequently, NPP and NCP can be assessed for any desired day-length and irradiance from single Φ_PSII_ measurements (assuming state-state conditions of the cellular light- acclimation processes). This conclusion is in accordance with current understanding of the light acclimation in microalgae, which is categorized in sequential ‘time windows’. The initial light acclimation processes happens within seconds to minutes (e.g. photochemical and non-photochemical quenching), whereas protein synthesis and pigment metabolism occurs on time scales of hours, while metabolic changes occurs on the time scales of generations [7,12,55]. Nymark et al. [55] have shown that despite complex short-term changes on levels of gene transcription, protein synthesis, and pigment metabolism, microalgae are able to secure a relative stable electron transfer rate through the PSII reaction centre for time period <12 hours. This has been explained as an evolutionary mechanism to secure an efficient photosynthetic capacity even under fluctuating light conditions. Changes in ETR, however, occurred after prolonged exposure to high light of >12 hours [51,55], which is why we recommend that light:dark shift incubations spanning 24 hours are carried out in a way to avoid light periods longer than 12h. It is beyond the scope of this work to elaborate on the photo acclimation processes in microalgae.

Comparing diel integrated rates of NPP showed a linear relationship between P*_PSII_ and P*_C_ with a slope coefficient of 1.4 (Fig 7A). Here we have applied the electron requirement for gross C fixation [7.6 mol é (mol C)^−1^] in the calculation of P*_PSII_ to compare with net C fixation rates. The resulting electron requirement for net C fixation of 1.4 times that of gross C fixation [*Κ*_C_ = 10.9 ± 1.1 mol é (mol C)^−1^ (R^2^ = 0.86)] accounts for the ‘costs’ for metabolic activity and cell maintenance, and includes the fraction of labeled ^14^C that recirculates between states of fixed (biomass) and respired (CO_2_) carbon. Estimates of NPP using variable fluorescence rely on the precision and the potential variability of *Κ*_C_. Here we estimated P_PSII_ from triplicated bottles; and in each bottle Φ_PSII_ was measured 4 to 6 times over the 24h incubation. Across the entire data set the coefficient of variance was 4.2 and 16.3% for low and high light, respectively. The 4 times higher variance at high light is related to the inherent decrease in the signal to noise ratio of Φ_PSII_ with increasing irradiance. The variability of a*_PSII_ is on the same order, while the precision of the irradiance measurements are much better (<1%) assuming careful measurements with a calibrated sensor. Thus, we conclude that at the applied settings the NPP was estimated from absolute PAM (Φ_PSII_) measurements with a ~20% accuracy.

As for the 2 hour incubation we recalculated *Κ*_C_ for the NPP without a spectral correction. This resulted in a value of 15.9 ± 1.6 mol é (mol C)^−1^, which corresponds to an overestimation of a factor 1.5 (Table 4). To our knowledge, no previous studies have compared rates of ETR_PSII_ and NPP obtained over 24h, thus the result has to be evaluated in context of the studies listed in Table 4.

Nutrients were not limiting in the current study (N > 1 μM in all samples, Table 1) and we rule out Φ_PSII_ to be depressed by nutrient limitations. The effect of nutrient limitations on Φ_PSII_ is, nevertheless, not consistent, but evidence of Φ_PSII_ depression due to N limitation seem convincing in some cases [56], however many studies point to only limited depressing of Φ_PSII_ during nutrient starvation, partly due to acclimation processes [22,26,57–59].

Like C fixation, NCP in O_2_ units (P*_O2_) resulted in a linear relationship with P*_PSII_, with a slope coefficient corresponding to an electron requirement of 6.5 ± 0.9 mol é (mol O_2_)^−1^. This was ~40% lower than for C fixation during 24 hours and supports the assumption of an considerable electron consumption related to the ‘dark’ reactions of C fixation, including cell maintenance and nutrient uptake. The respiratory O_2_ consumption explain the majority of the discrepancy between the empirical *Κ*_C_ and the theoretical one [4 é (mol O_2_)^−1^], and accounted to 10.1 ± 5.2% and 44.9 ± 19.3% of the NPP during high and low light, respectively. The rates of respiration are consistent with typical ratios published for pelagic ecosystems [60,61]. The slope coefficient of P_O2_ versus P_C_ was 1.6 ± 0.5, which complies with the resolved PQ of 1.4 in the current study [50].

The Arctic marine environment is experiencing dramatic changes in sea ice cover, terrestrial run-off and light attenuation [62]. It is expected that these changes will affect primary production. Yet most routine monitoring programs of marine ecosystems are limited to few measurements of primary production, as the benchmark ^14^C method is time consuming and expensive. Application of variable fluorescence techniques has the potential to expand routine measurements to larger regions and with higher temporal resolution. This, however, will require detailed and reproducible assessment of the electron requirement for carbon fixation under natural variable conditions [16,63]. Not least in the under-sampled Arctic [62]. Future studies should ideally compare *in situ* measured variable fluorescence with *in situ* bottle incubations of C fixation and O_2_ production to preserve the natural optical properties of phytoplankton absorption and available spectral irradiance.
